# The use of TASER devices in UK policing: an update for clinicians following the recent introduction of the TASER 7

**DOI:** 10.1136/emermed-2022-212521

**Published:** 2022-07-19

**Authors:** Anthony Bleetman, Alan E Hepper, Robert D Sheridan

**Affiliations:** 1 Emergency Department, Beilinson Hospital, Petah Tikva, Israel; 2 DSTL, Salisbury, Wiltshire, UK

**Keywords:** assessment, violence, trauma, pre-hospital, Forensic Medicine

## Abstract

Taser was introduced into UK policing in 2003 to bridge the operational gap between use of incapacitant sprays and firearms. Use of force reporting in the UK indicates that Taser is relatively safe provided that it is used lawfully. Taser use can result in injuries and has been implicated in a small number of deaths. The latest version of the weapon, the TASER 7, has entered UK policing. The TASER 7 uses a novel probe that has implications for the medical community. A review of Taser medical effects and probe removal for TASER 7 are presented.

Conducted energy devices (CEDs), also known as conducted electrical weapons, are hand-held electronic devices widely used by law enforcement agencies in the USA, Canada, the UK, Europe, Australia and elsewhere. Together with less-lethal impact rounds, CEDs are designed to bridge the gap between close quarter options, such as batons and irritant sprays, and lethal firearms in helping to resolve serious and violent incidents.

CEDs may be used in a number of ways to de-escalate incidents. Drawing the device from its holster, aiming it at a subject, producing a high-voltage arc display at the front of the device (‘arcing’) or painting a laser aiming dot on the subject may all have deterrent value.^1^ As a last resort, however, CEDs may be used to administer an electrical discharge to a subject. The discharge, which takes the form of brief, repetitive pulses, may be delivered either by direct application of the front end of the weapon to the subject (drive-stun) or by firing two electrically tethered projectiles (probes) of opposite polarity up to a distance of 21–25 feet, depending on the model. The front end of each probe is designed to penetrate the skin or to attach to an individual’s clothing. By far the vast majority of uses in which people are subjected to electrical discharge involve the firing of probes.^1^

When probes are fired from the cartridge at the front end of the CED, the top probe flies towards the subject in line with the ‘muzzle’ of the CED, while the lower probe diverges downward. This means that the spread of the probes increases as they approach their target. The spread of the probes is required for the discharge to exert its desired effect.^1^

CED discharge has two principal effects on the subject. First, in ‘drive-stun’ mode, or when the probes are narrowly spaced, the discharge results in pain; second, when the fired probes are more than about 9 inches (23 cm) apart, the subject will experience pain and so-called neuromuscular incapacitation, stiffening up and falling to the ground, unable to maintain voluntary control of their movement.

The most common brand of CED is manufactured by Axon Enterprise, Inc, under the TASER trademark. Devices include the TASER X26 (launched by Axon in 2003 and discontinued in 2014), the TASER X2 (launched in 2011) and the TASER 7 (launched in late 2018).

While there are differences in the electrical discharge output of the various TASER devices, a major distinction between the TASER X26 and the later TASER devices is that the TASER X26 has a single cartridge, while the TASER X2 and TASER 7 have twin cartridges. The twin cartridge devices offer the user a rapidly deployable second shot back-up in the event of a probe miss on the first shot.

The TASER 7, which will progressively replace the older devices, diverges from both older devices due to its novel probe design.

## CED use from a UK perspective

Home Office statistics for England and Wales indicate that police officers fired probes on nearly 3250 occasions in the 12-month period to March 2021.^2^ In contrast, drive-stun was used less than 80 times during the same period.^2^

Since their introduction into UK policing in 2003, the use of CEDs has been associated with non-fatal injuries and a small number of deaths, with one death being attributed solely to CED use. In this case, the electrical discharge from the CED ignited fuel that the subject had poured on himself.^2^ There have also been a small number of fatal outcomes in the UK where the electrical discharge from the CED was considered to have played a contributory role.^3–8^

Considering that probes have been deployed on more than 26 000 occasions in the 4 years to March 2021, CEDs appear to be relatively safe weapons provided that they are used lawfully by the police in situations in which less injurious options for use of force are inappropriate or unlikely to succeed in resolving a violent encounter. There remains a paucity of prospective studies on their use in the operational setting and it is unclear whether certain vulnerabilities increase the risk of morbidity and mortality.

In the UK, the medical effects of CEDs are considered by the Scientific Advisory Committee on the Medical Implications of Less-Lethal Weapons (SACMILL), a government-sponsored independent advisory committee. SACMILL assesses the medical impacts of less-lethal weapons, including CEDs, used by police in the UK, and makes recommendations regarding their use.

## Medical effects of CED discharge

The deployment of probes can be expected to cause small electrical burns around the skin contact sites and could be followed by secondary injury from uncontrolled falls^3^ which are among a number of injury mechanisms that have been identified.^3 9^

Partly because of the range of potential injuries that have been associated with CED discharge, the UK Faculty of Forensic and Legal Medicine (FFLM), together with other bodies representing UK healthcare practitioners, have recently introduced guidance and specialist training around the effects of CEDs and the medical assessment of those in police custody who have been exposed to CED discharge.^10^ For patients attending the ED after exposure to CED, they should undergo an ECG, and cardiac monitoring should be considered in patients with a significant cardiac risk or pacemaker (although delayed dysrhythmias are thought to be extremely rare). Injuries may be present that were sustained prior to CED discharge and therefore a thorough clinical assessment should be undertaken. Specialist assistance might be necessary for the removal of CED barbs in sensitive body areas. Pregnant patients are advised to undergo obstetric review following exposure to CED. Perhaps the most important aspect of assessment and management of these patients in the ED relates to the assessment of acute behavioural disturbance, acute psychosis or drug-related issues that led to the circumstances in which CED was deployed.

## CED probes

Where the probes of the TASER X26 and TASER X2 have penetrated the skin in an uncomplicated anatomical region, they may be removed by tensing the skin around the probe-penetration site and tugging firmly on the body of the probe. The removal of probes that have penetrated sensitive areas, or have become entrapped in bone, may require the attention of specialist medical staff and it is then that patients may present to the ED. Sensitive areas where probe injuries have been described include the eyes, genital area, mouth, neck and head.

**Figure 1:**
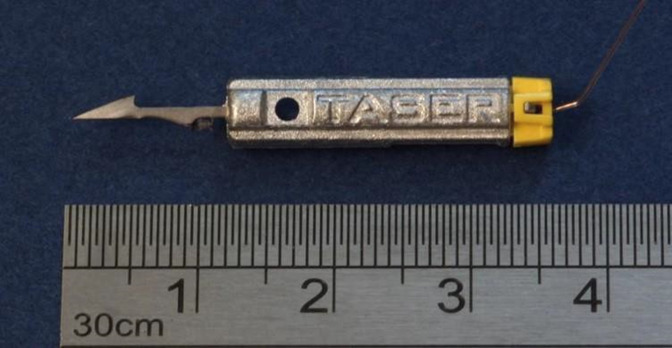
TASER X2 probe: a one-piece projectile.

The probes of the new TASER 7 device differ from previous models in a number of ways, but the key change for clinicians lies in the two-part construction of the probes. Earlier versions of TASER CEDs have employed solid, one-piece probes in which the shaft is attached to the probe body (figure 1).

**Figure 2:**
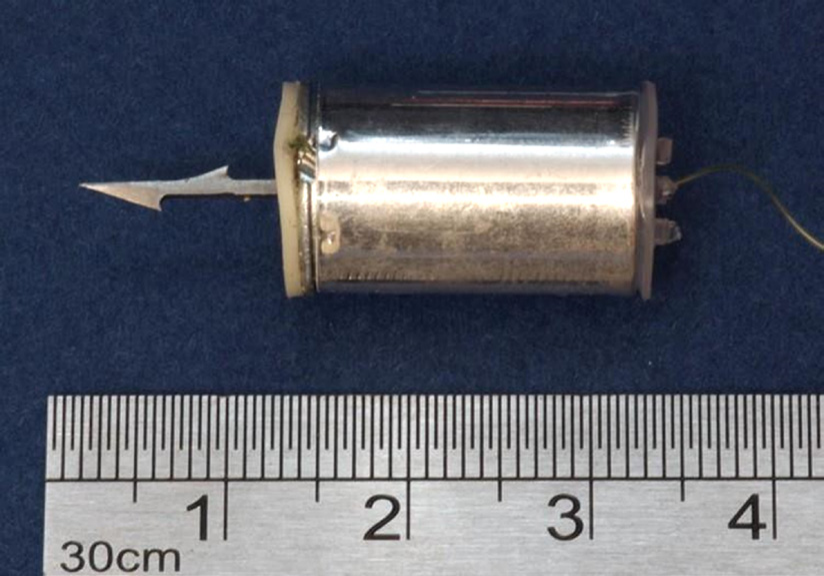
TASER 7 probe—intact.

**Figure 3:**
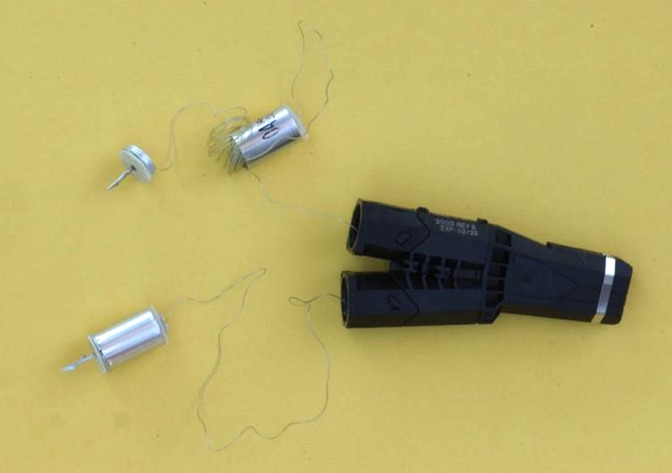
TASER 7 probe separated (upper) and intact (lower).

**Figure 4:**
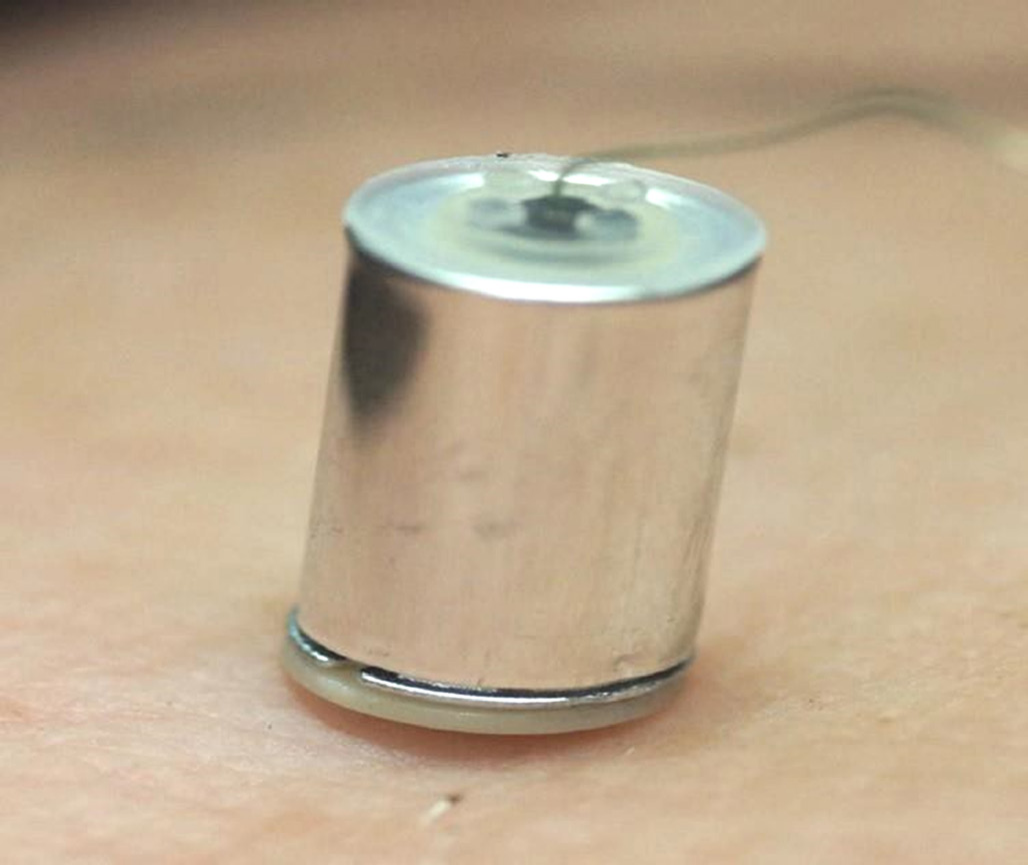
An intact TASER 7 probe with its shaft fully embedded in tissue.

**Figure 5:**
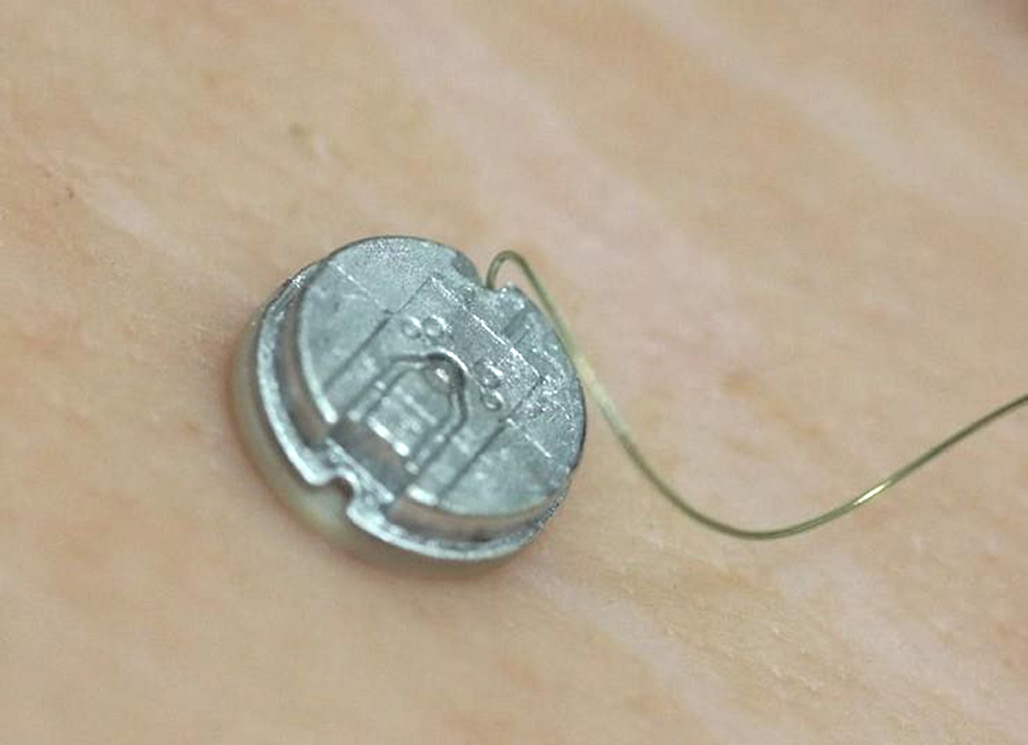
Tissue-embedded TASER 7 probe after separation of the cylindrical probe body from the shaft-bearing frontal assembly.

In the new TASER 7 device, the probe shaft is fixed to a base plate which sits on the end of a cylinder from which the stored cable pays out during flight. At its front end, the cylinder is attached to the front assembly at six crimp points around the circumference of the base plate. The base plate/shaft assembly can detach from the cylinder during flight and on impact. Figures 2–5 illustrate the probes.

The mass and velocity, and hence the kinetic energy and momentum, of the TASER 7 probe is greater than that of its predecessors. This means that the probe shaft may embed deeper into tissue, even though the barbed point is of the same length.

**Figure 6:**
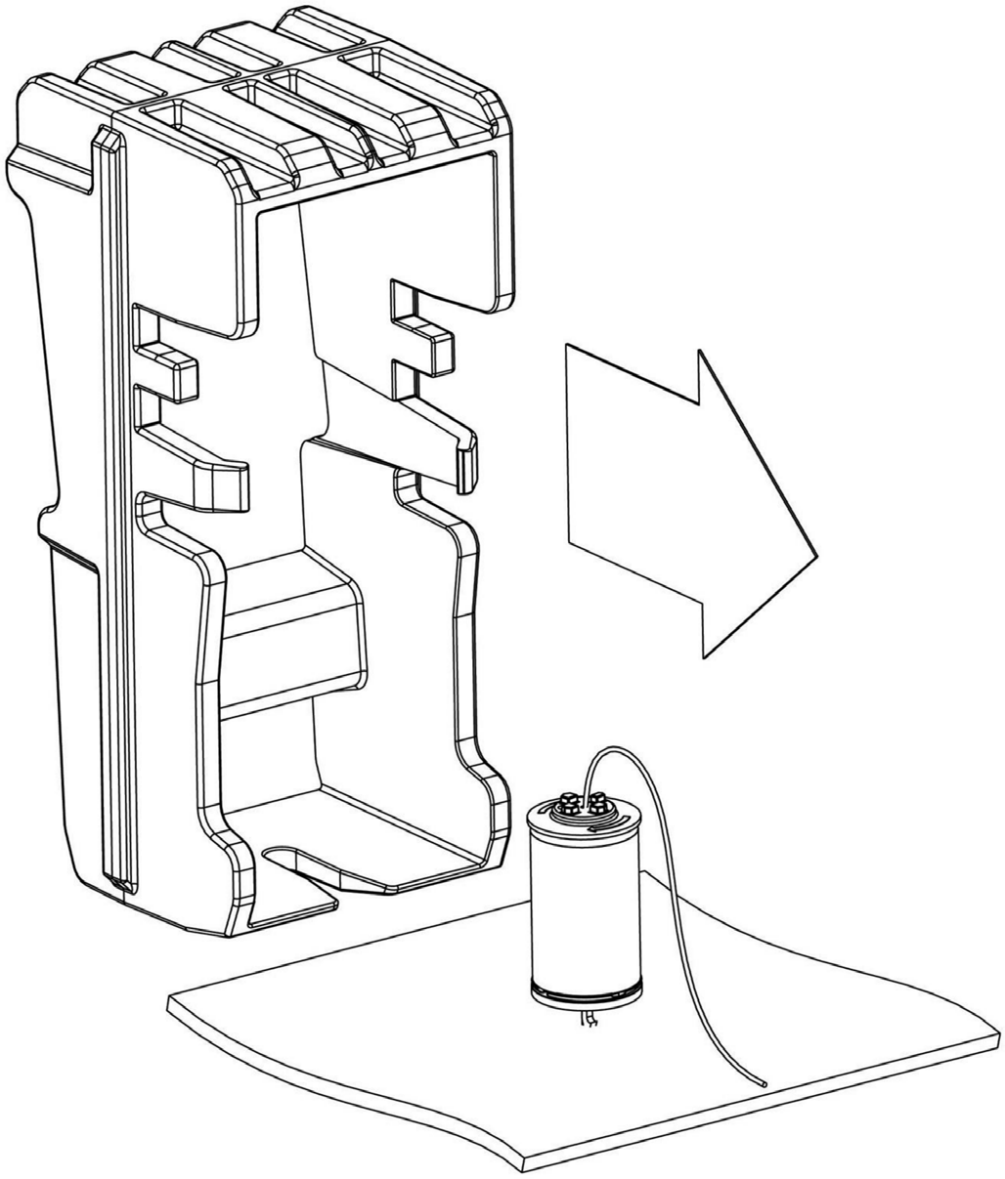
Probe removal tool.

Unlike its predecessors, skin-embedded TASER 7 probes should not be removed by simply tugging the probe body. Instead, police officers are issued with a plastic tool specifically designed for the purpose of probe removal. Because the tool is dual purpose—it also serves as the carrier used to store unused cartridges—it should be readily available from police officers in hospital settings on request (figure 6). An alternative probe removal tool, which operates on the same principle as the cartridge carrier, is marketed by the manufacturers of the TASER 7.

**Figure 7:**
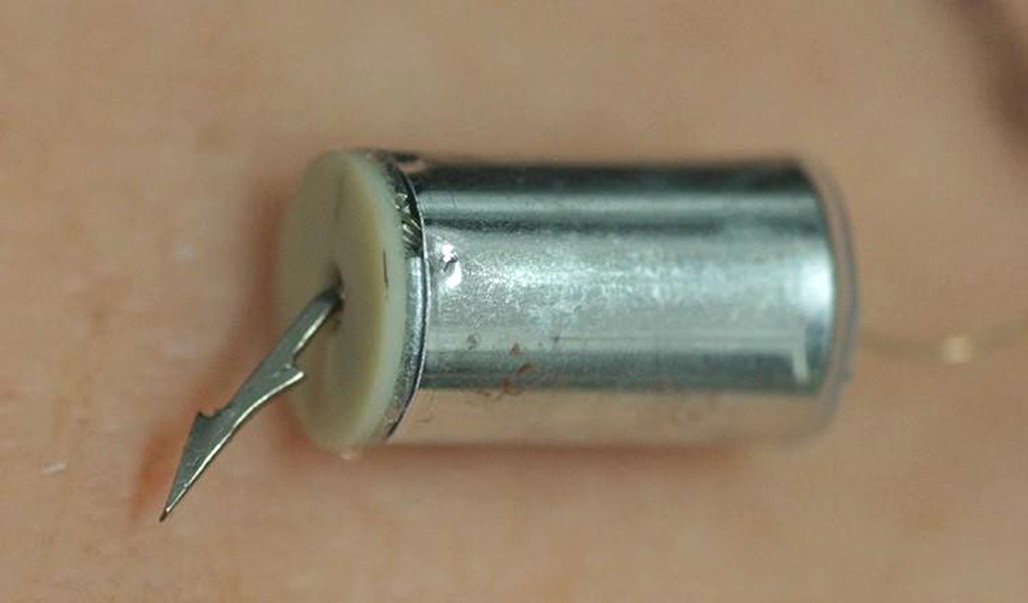
TASER 7 probe showing the deformed shaft after striking an underlying rib.

We conducted a small study using an animal model to assess different methods of removal of tissue-embedded TASER 7 darts.^10^ The probes were fired into a section of porcine thorax/abdomen obtained from an animal that had been humanely slaughtered at a licensed abattoir. Removal of embedded probes using a needle holder from a standard suture pack was technically far more difficult than using the police-issued removal tool: using a needle holder also made it more difficult to produce an axial force on the probe, resulting in the shaft twisting in the underlying tissue and the potential for increasing tissue damage. We also identified that the shaft of the TASER 7 probe may bend on impact with underlying bone and that the probe body can separate into its two component parts on impact or in flight (figure 7).

CEDs are now routinely used by UK Police and can cause effects of which the emergency clinician should be aware, in particular the differences relating to the new TASER 7.

Key recommendations are listed in box 1.

Box 1 **Recommendations for clinicians**

1. The plastic tool issued to police officers should be used to remove a tissue-embedded probe in non-sensitive areas of the body. This was found to be easier than using a needle holder from a suture pack. A TASER 7 police officer should be able to provide the removal tool to medical staff.
2. A deformed barb may require removal by a medical professional in hospital as it may not come out easily with the plastic tool.
3. Probes in particularly vulnerable areas (eg, the eyes, head, neck or genitalia) should always be removed by medical professionals only, ideally in a hospital setting.
4. Clinicians need to be aware of the potential for secondary injuries following CED deployment.
5. Clinicians need to consider any medical, toxicological or psychiatric conditions that preceded CED deployment (particularly acute behavioural disturbance-excited delirium).

CED, conducted energy device.

Further information on potential probe removal methods may be found on the FFLM website along with more detailed information on the clinical effects of CEDs.^11^
